# Impact of Age and Gender on the Prevalence and Prognostic Importance of the Metabolic Syndrome and Its Components in Europeans. The MORGAM Prospective Cohort Project

**DOI:** 10.1371/journal.pone.0107294

**Published:** 2014-09-22

**Authors:** Julie K. K. Vishram, Anders Borglykke, Anne H. Andreasen, Jørgen Jeppesen, Hans Ibsen, Torben Jørgensen, Luigi Palmieri, Simona Giampaoli, Chiara Donfrancesco, Frank Kee, Giuseppe Mancia, Giancarlo Cesana, Kari Kuulasmaa, Veikko Salomaa, Susana Sans, Jean Ferrieres, Jean Dallongeville, Stefan Söderberg, Dominique Arveiler, Aline Wagner, Hugh Tunstall-Pedoe, Wojciech Drygas, Michael H. Olsen, on behalf of the MORGAM Project

**Affiliations:** 1 Department of Internal Medicine, Glostrup Hospital, University of Copenhagen, Glostrup, Denmark; 2 Research Centre for Prevention and Health, Glostrup Hospital, University of Copenhagen, Glostrup, Denmark; 3 Division of Cardiology, Holbæk University Hospital, Holbæk, Denmark; 4 Cerebro and Cardiovascular Epidemiology Unit, National Centre of Epidemiology Surveillance and Health Promotion, National Institute of Health, Rome, Italy; 5 UKCRC Centre of Excellence for Public Health Research (NI), The Queen's University of Belfast, Belfast, Northern Ireland; 6 Clinica Medica e Istituto Auxologico Italiano, Monza, Milano, Italy; 7 Research Centre on Public Health, University of Milano Bicocca, Monza, Italy; 8 National Institute for Health and Welfare (THL), Helsinki, Finland; 9 Department of Health, Barcelona, Spain; 10 Department of Cardiology, Toulouse University School of Medicine, Rangueil Hospital, Toulouse, France; 11 Institut Pasteur de Lille, Lille, France; 12 Department of Public Health and Clinical Medicine, Cardiology and Heart Centre, Umeå University, Umeå, Sweden; 13 Department of Epidemiology and Public Health, University of Strasbourg, Faculty of Medicine, Strasbourg, France; 14 Cardiovascular Epidemiology Unit, University of Dundee, Ninewells Hospital and Medical School, Dundee, United Kingdom; 15 Department of Epidemiology, CVD Prevention and Health Promotion, National Institute of Cardiology, Warsaw, Poland; 16 Department of Endocrinology, Center of Individualized Medicine in Arterial Diseases (CIMA), Odense University Hospital, Odense, Denmark, and Hypertension in Africa Research Team (HART), North-West University, Potchefstroom, South Africa; Azienda Ospedaliero-Universitaria Careggi, Italy

## Abstract

**Objective:**

To investigate the influence of age and gender on the prevalence and cardiovascular disease (CVD) risk in Europeans presenting with the Metabolic Syndrome (MetS).

**Methods:**

Using 36 cohorts from the MORGAM-Project with baseline between 1982–1997, 69094 men and women aged 19–78 years, without known CVD, were included. During 12.2 years of follow-up, 3.7%/2.1% of men/women died due to CVD. The corresponding percentages for fatal and nonfatal coronary heart disease (CHD) and stroke were 8.3/3.8 and 3.1/2.5.

**Results:**

The prevalence of MetS, according to modified definitions of the International Diabetes Federation (IDF) and the revised National Cholesterol Education Program-Adult Treatment Panel III (NCEP-ATPIII), increased across age groups for both genders (*P*<0.0001); with a 5-fold increase in women from ages 19–39 years to 60–78 years (7.4%/7.6% to 35.4%/37.6% for IDF/NCEP-ATPIII) and a 2-fold increase in men (5.3%/10.5% to 11.5%/21.8%). Using multivariate-adjusted Cox regressions, the associations between MetS and all three CVD events were significant (*P*<0.0001). For IDF/NCEP-ATPIII in men and women, hazard ratio (HR) for CHD was 1.60/1.62 and 1.93/2.03, for CVD mortality 1.73/1.65 and 1.77/2.06, and for stroke 1.51/1.53 and 1.58/1.77. Whereas in men the HRs for CVD events were independent of age (MetS*age, *P*>0.05), in women the HRs for CHD declined with age (HRs 3.23/3.98 to 1.55/1.56; MetS*age, *P* = 0.01/*P* = 0.001 for IDF/NCEP-ATPIII) while the HRs for stroke tended to increase (HRs 1.31/1.25 to 1.55/1.83; MetS*age, *P*>0.05).

**Conclusion:**

In Europeans, both age and gender influenced the prevalence of MetS and its prognostic significance. The present results emphasise the importance of being critical of MetS in its current form as a marker of CVD especially in women, and advocate for a redefinition of MetS taking into account age especially in women.

## Introduction

Since Reaven in 1988 [1] established the clinical importance of the clustering of the metabolic disorders dysglycemia, central adiposity, hypertension and dyslipidemia (low levels of high density lipoprotein cholesterol (HDL-C) and high levels of triglycerides), known as the metabolic syndrome (MetS), many studies [2]–[22] have shown a higher risk of cardiovascular disease (CVD) in the presence of MetS. However, in recent years the clinical relevance of MetS in assessing risk for developing CVD has been questioned since studies [15], [23]–[26] have stated that MetS is no single disease entity and no better than its individual components in identifying individuals at high risk of CVD. This critical appraisal of MetS as a prognostic marker of CVD risk comes at a time when the prevalence of MetS has increased dramatically, with approximately one-fourth of the adult population in Europe carrying this syndrome [27]. Therefore, it is important to clarify whether the utility of MetS can be optimized as a screening tool for identifying individuals at high risk of CVD.

From previous European work there is some indication of age and gender interactions on the association between MetS and CVD risk since the risk of CVD in the presence of MetS was more strongly related to women than men [17]–[22] and to older individuals [28]. However, despite this well described influence of gender and age on MetS, only few national studies have investigated age and gender specific MetS prevalence [5], [8]–[9], [11], and none of the studies looked at the impact of age and gender on the prognostic significance of MetS. Furthermore, if the purpose of MetS is to shift emphasis to earlier intervention with for example lifestyle changes, it is crucial to get a better understanding of the impact of age and gender on its prevalence and prognostic significance in a more representative sample, and therefore, studies incorporating a broader range of study participants are needed.

Using the MORGAM Project, which consists of large European population-based cohorts of men and women aged 19–78 years, with uniform standardized data collection, we aimed to investigate the influence of age and gender on the prevalence and prognostic significance of MetS in regard to fatal and nonfatal CHD, fatal and nonfatal stroke, and CVD mortality, defined by the National Cholesterol Education Program – Adult Treatment Panel (NCEP-ATP III) criteria [29] and the International Diabetes Federation (IDF) criteria [30].

## Methods

### Cohorts

The present study used baseline (1982–1997) and follow-up data on subsequent cardiovascular events from 36 cohorts in 10 European countries from the MOnica, Risk, Genetics, Archiving and Monograph (MORGAM)-Project [31] (table S1; see supporting information). As seen in table S1, age at baseline, gender composition as well as the follow-up time differed among the countries.

The cohorts in the MORGAM Project had either been a part of the World Health Organization's MONICA Project or had used the same standardized MONICA survey procedures for data collection as described in the MORGAM-manual [32]. Exclusion criteria at baseline included a history of stroke (ischemic or hemorrhagic; n = 969) or CHD (myocardial infarction, angina pectoris, coronary artery by-pass graft, or coronary angioplasty; n = 2899), and missing values on (1) any of the MetS components: blood pressure (BP), use of antihypertensive drugs, serum high density lipoproteins cholesterol (HDL-C), body mass index (BMI), diabetes (n = 4057) and serum triglycerides (n = 1586); and (2) potential confounders such as smoking and total serum cholesterol (n = 167), leaving a total of 69094 participants for analyses.

The use of antihypertensive drugs, daily smoking, and diabetes at baseline were self-reported. BMI was calculated as weight (kg) divided by the square of the height (m^2^). Weight was measured with weight balance scales, and height with a stadiometer. Waist circumference (WC) was reported in cm. BP was measured twice in the right arm in the sitting position using a standard or random zero mercury sphygmomanometer after a 5-minute rest [33] except in five cohorts where BP was measured only once. The mean of the first and second systolic BP (SBP) and diastolic BP (DBP) was used when possible. Total cholesterol, HDL-C, and triglycerides were measured in serum samples by local laboratories [33]. Since fasting levels differed between cohorts, a categorized fasting variable was used as adjustment in the Cox model: (1) full fasting: overnight/at least 8 hours of fasting before blood sampling; (2) semi-fasting: between 4–8 hours of fasting; and (3) non-fasting: less than 4 hours of fasting.

As endpoints we chose fatal and nonfatal CHD and stroke, respectively, in order to have statistical power to identify possible endpoint related differences. Furthermore, we chose CVD mortality due to the greater validity of fatal events, as well as to include other clinical manifestations of CVD, besides CHD and stroke, that are also of atherosclerotic origin. Observations continued until death or the end of a fixed follow-up period (December 31^st^ 1998- December 31^st^ 2007 depending on the cohort). The mean follow-up time was 12.2 years. Fatal cases were identified by national or regional health information systems. In most cohorts, nonfatal cases were identified by hospital discharge registers. Most MORGAM centres used the WHO MONICA diagnostic criteria [33] to validate the events occurring during follow-up. Details including quality assessments of MORGAM endpoints and baseline data have been described previously [34]–[35].

### Classification of Metabolic Status

We used modified versions of MetS according to both the IDF criteria [30] and the 2004 revised NCEP-ATP III criteria [29]. In order to maximize sample size, BMI was used in the main analyses; analyses were also replicated using WC. A scatter plot was drawn to find the BMI cut-offs which corresponded to WC with specific reference to a European population. Furthermore, since data on plasma glucose was not available, the self-reported presence of diabetes or use of anti-diabetic drugs was used instead. According to the IDF criteria, MetS was based on the presence of a BMI≥30 kg/m^2^ in men and ≥25 kg/m^2^ in women and 2 or more of the following components: (1) BP≥130 mmHg (systolic) or ≥85 mmHg (diastolic) or use of antihypertensive drugs; (2) triglyceride ≥1.7 mmol/l; (3) HDL-C <1.03 mmol/l in men and <1.29 mmol/l in women; and (4) the presence of diabetes or use of anti-diabetic drugs. According to the NCEP-ATP III criteria, MetS was based on the presence of 3 or more of the 5 criteria which are identical to those provided by IDF. With specific reference to a European population, the cut-offs for WC was ≥94 cm in men and ≥80 cm in women according to the IDF criteria, and >102 cm in men and >88 cm in women according to the NCEP-ATP III criteria.

### Statistical Analyses

Statistical Analysis Software (SAS Institute Inc, Cary, NC) version 9.2 was used for all analyses. Baseline characteristics were presented as median and the 5^th^ and 95^th^ percentiles or as percent. Discrete variables were compared using the Chi-square test, while continuous variables were compared using Student's t-test or non-parametric Mann-Whitney test, according to the normality of variables. Differences in continuous variables between groups were tested using one-way ANOVA. Due to differences in cohort follow-up time, the incidence rates of cardiovascular events per 1000 person years were reported instead of absolute risk for MetS and its individual components.

Since age distribution varied among the populations, the prevalence of MetS at baseline was presented for a fixed age-interval of 50–59 years, allowing for a meaningful comparison between the populations since this age range was covered by all the populations. Furthermore, due to significant interactions between age and gender for the baseline prevalence of MetS and its components using logistic regression models adjusted for country, total cholesterol, smoking- and fasting status (all *P*<0.0001, table 1), separate analyses were carried out for men and women in various baseline age groups: 19–39 years, 40–49 years, 50–59 years, and 60–78 years. Although interactions between country and age as well as country and gender were also significant for the prevalence of MetS and most of its components, regression analyses were not carried out separately for men and women within each country due to lack of statistical power. Instead, the Cox proportional hazard model was stratified by country allowing for the baseline hazard to vary between countries. In addition, we adjusted for age, total cholesterol, smoking and fasting status, and used time from baseline as the time scale. Hazard ratios (HRs) and 95% confidence intervals (95% CIs) for CHD, stroke, and CVD mortality in relation to the different definitions of MetS were estimated separately for men and women of various age groups. Only the interaction between MetS and age for women with regards to CHD risk was significant (table 2). In the outcome analysis, for participants who experienced multiple events we only considered the first.

**Table 1:** Significance of age, gender, country, and their interactions, for risk of having the metabolic syndrome or its individual components in adjusted logistic regression models.

Influence of:	age	gender	country	age* gender	country* gender	country* age	country* gender*
*P*-values:							age
MetS ATP	<0.0001	<0.0001	<0.0001	<0.0001	<0.0001	<0.0001	0.41
MetS IDF	<0.0001	<0.0001	<0.0001	<0.0001	<0.0001	<0.0001	0.74
Individual MetS Components (dichotomized):							
Blood Pressure	<0.0001	<0.0001	<0.0001	<0.0001	<0.0001	<0.0001	0.88
Body Mass Index	<0.0001	<0.0001	<0.0001	<0.0001	<0.0001	<0.0001	0.009
Triglycerides	<0.0001	<0.0001	<0.0001	<0.0001	<0.0001	0.0005	0.004
HDL-Cholesterol	<0.0001	<0.0001	<0.0001	<0.0001	<0.0001	<0.0001	<0.0001
Diabetes	<0.0001	0.09	0.45	<0.0001	0.10	0.002	0.15

*P*<0.05 indicates a significant interaction term in the logistic regression model.

Adjusted for age, sex, country, total cholesterol, smoking- and fasting status.

MetS ATP indicates the metabolic syndrome defined by the National Cholesterol Education Program - Adult Treatment Panel III; and MetS IDF, the metabolic syndrome defined by the International Diabetes Federation.

**Table 2:** Significance of interactions between age, gender, and the metabolic syndrome for subsequent CVD risk in adjusted Cox regression models.

	Fatal and nonfatal		
	CHD	Stroke	CVD mortality
Interactions:	*P*-values:	*P*-values:	*P*-values:
sex*MetS IDF	0.07	0.97	0.90
sex*MetS ATP	0.005	0.32	0.05
age*MetS IDF	0.004	0.19	0.02
age*MetS ATP	0.0003	0.56	0.01
**Men**			
age*MetS IDF	0.30	0.91	0.10
age*MetS ATP	0.53	0.13	0.16
**Women**			
age*MetS IDF	0.01	0.86	0.18
age*MetS ATP	0.001	0.56	0.05

*P*<0.05 indicates a significant interaction term in the Cox regression model.

CHD indicates coronary heart disease; CVD, cardiovascular disease; MetS ATP, the metabolic syndrome defined by the National Cholesterol Education Program - Adult Treatment Panel III; and MetS IDF, the metabolic syndrome defined by the International Diabetes Federation.

Cox model adjusted for smoking (yes/no), cholesterol (continuous), and fasting (full/semi/no fasting).

All explanatory variables met the proportional hazards assumption of the Cox regression model, assessed by Schoenfeld residuals. The linearity of the continuous variables was assessed using quadratic and cubic effects as well as cubic splines (a piecewise fitting of polynomial equations).

Analyses were replicated in subsets using WC instead of BMI, and using a reduced dataset excluding participants in antihypertensive treatment and non-fasting or semi-fasting participants.

For all analyses a 2-tailed *P*<0.05 was considered statistically significant.

## Results

### Risk Factors

**Figure 1:**
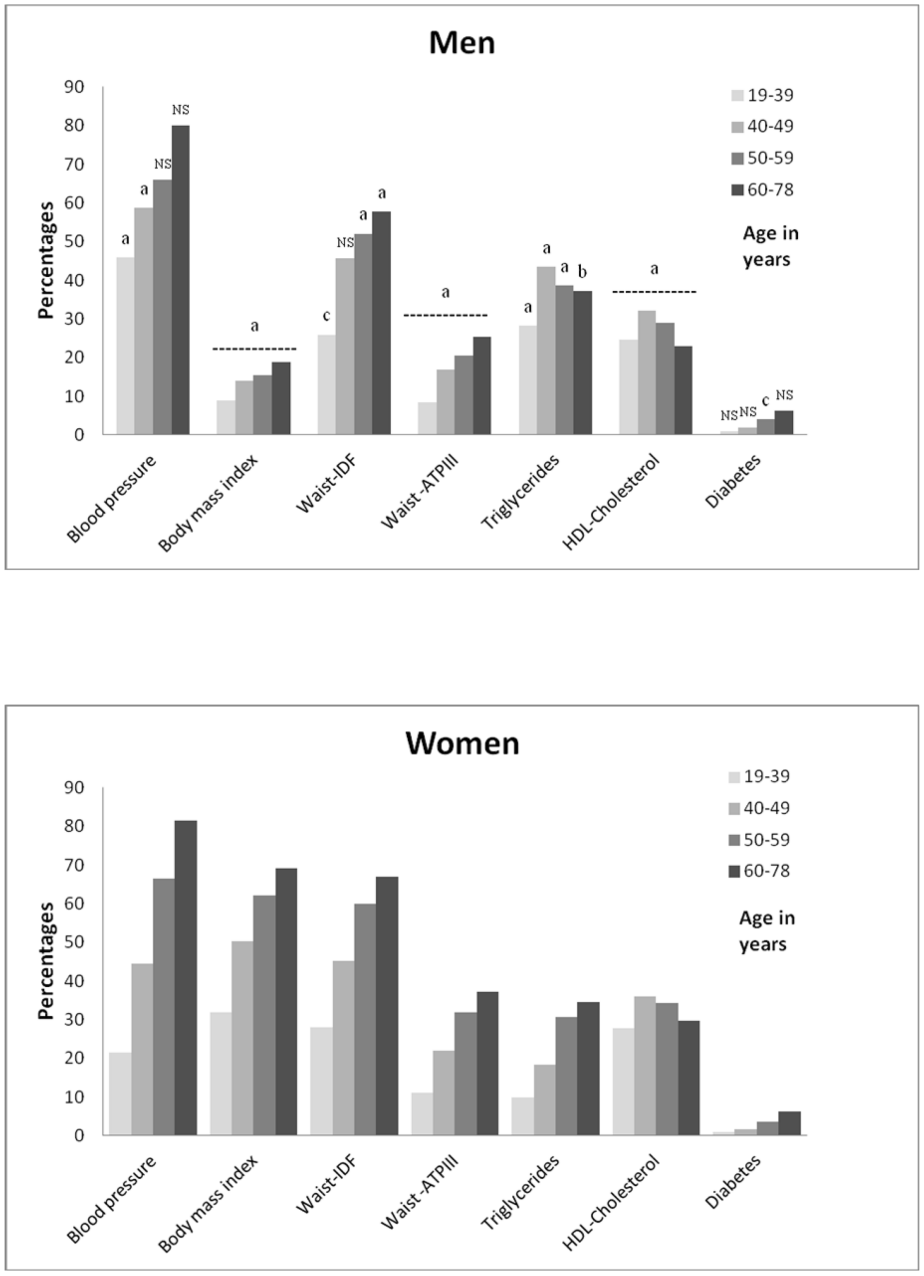
Frequency of each MetS component according to gender and age group. MetS indicates the metabolic syndrome; IDF, the International Diabetes Federation; and ATPIII, Adult Treatment Panel III. The men/women ratio is 38639/30455 for all components except for waist circumference (men/women  = 23817/152899) since not all cohorts registered waist circumference. *P*<0.0001 for each MetS component across age groups in men and women separately. Gender differences within each age group, with *P*<0.0001^a^, *P*<0.01^b^, *P*<0.05^c^, NS indicates non-significance, *P*>0.05, and the dashed line indicates the same level of significance across the age groups.

For both genders, the median values of the baseline metabolic risk factors as well as the prevalence of the individual components of MetS, except HDL-C, and triglycerides in men, increased across age groups (all *P*<0.0001; table 3 and figure 1, respectively). Age influenced the pattern of the MetS components in men and women, such that young women had a higher prevalence of obesity and low HDL-C, while younger men had a higher prevalence of elevated BP and elevated triglycerides. In older men and women, high BP was the most prevalent component of MetS.

**Table 3:** Distribution of risk factors in men (a) and women (B) according to age group.

(A) Men	Age				
	19–78 Years	19–39 Years	40–49 Years	50–59 Years	60–78 Years
Risk Factors					
Number of subjects	38639	8143 (21.1)	8409 (21.8)	17714 (45.8)	4373 (11.3)
Smoker	13463 (34.8)	3559 (43.7)^b^	3475 (41.3)^b^	5051 (28.5)	1378 (31.5)
Diabetes mellitus	1240 (3.2)	73 (0.9)	165 (2.0)	730 (4.1)	272 (6.2)
Antihypertensive treatment	3346 (8.7)	87 (1.1)	419 (5.0)	2053 (11.6)	787 (18.0)
BMI, kg/m^2^	26.0 (20.9–32.7)	24.8 (20.3–31.6)	26.0 (21.0–32.7)	26.3 (21.3–32.8)^b^	26.6 (21.1–33.4)^b^
Waist circumference^a^, cm	93 (77.5–111)	87 (74–107)	92.5 (77.5–111)	94 (79–112)	96 (79–114)
Total cholesterol, mmol/L	5.61 (4.01–7.53)	5.20 (3.70–7.20)	5.70 (4.10–7.70)^d^	5.74 (4.20–7.58)^d^	5.80 (4.20–7.70)
Triglycerides, mmol/L	1.42 (0.66–3.86)	1.21 (0.58–3.46)	1.54 (0.67–4.31)	1.47 (0.71–3.85)^b^	1.43 (0.70–3.66)^b^
HDL cholesterol, mmol/L	1.19 (0.77–1.85)	1.21 (0.81–1.81)^c^	1.16 (0.73–1.83)	1.19 (0.76–1.86)^c^	1.24 (0.81–1.94)^c^
Systolic blood pressure, mmHg	131 (107–170)	124 (105–150)	128 (106–161)	133 (108–172)	144 (112–188)
Diastolic blood pressure, mmHg	83 (65–104)	79 (61–98)	83 (66–104)	84 (67–105)	85 (67–106)

(B) Women	Age				
	19–78 Years	19–39 Years	40–49 Years	50–59 Years	60–78 Years
Risk Factors					
Number of subjects	30455	8799 (28.9)	8818 (29.0)	8560 (28.1)	4278 (14.1)
Smoker	8171 (26.8)	2780 (31.6)^b^	2630 (29.8)^b^	2032 (23.7)	729 (17.0)
Diabetes mellitus	810 (2.7)	87 (1.0)	154 (1.8)	303 (3.5)	266 (6.2)
Antihypertensive treatment	2903 (9.5)	104 (1.2)	525 (6.0)	1256 (14.7)	1018 (23.8)
BMI, kg/m^2^	25.1 (19.6–35.5)	23.2 (18.9–32.3)	25.0 (35.0–19.9)	26.4 (20.4–36.5)	27.4 (20.5–37.3)
Waist circumference^a^, cm	78.5 (65–103)	74 (63.5–96)	78.5 (66–102.5)	82 (68–107)	84.5 (68.5–107)
Total cholesterol, mmol/L	5.59 (4.00–7.73)	4.90 (3.70–6.70)	5.40 (4.00–7.30)	6.10 (4.50–8.20)	6.30 (4.60–8.30)
Triglycerides, mmol/L	1.11 (0.56–2.84)	0.90 (0.50–2.14)	1.06 (0.55–2.71)	1.32 (0.66–3.20)	1.40 (0.72–3.28)
HDL cholesterol, mmol/L	1.44 (0.93–2.14)	1.46 (0.98–2.12)^d^	1.41 (0.90–2.10)	1.43 (0.91–2.16)	1.47 (0.93–2.23)^d^
Systolic blood pressure, mmHg	126 (101–170)	116 (98–140)	124 (101–160)	135 (107–178)	145 (113–185)
Diastolic blood pressure, mmHg	79 (61–100)	73 (58–91)	79 (63–100)	83 (65–104)^c^	83 (66–104)^c^

Values are expressed as numbers (percentages) or median (5% to 95% percentiles).

a Number of subjects  = 39106 (Men/Women  = 23817/15289) since not all cohorts registered waist circumference.

Comparison of risk factor values between age groups (i.e., 19–39 years versus 40–49 years, 40–49 years versus 50–59 years, and so on) for men and women separately, all *P*<0.0001, except. ^b^ *P*<0.01, ^c^ *P*<0.05, and ^d^ *P*>0.05.

Comparison of risk factor values between men and women within each age group, all *P*<0.0001, except for SBP levels in ages 60–78 years, and % of diabetics in ages 19–49 years and ages 60–78 years, where all *P*>0.05.

### Metabolic Syndrome

Despite comparing men and women separately, and using a fixed age interval of 50–59 years covered by all the populations, baseline MetS prevalence, defined by IDF/NCEP-ATP III, still varied greatly among populations (5.0–18.1%/10.8–34.5% in men and 11.3–45.0%/12.6–46.1% in women; table S1, see supporting information) leading to country interactions independently of differences in age and gender (table 1).

In the total population, there was a higher prevalence of MetS when using the NCEP-ATP III criteria compared to the IDF. This difference between IDF and NCEP-ATP III was more pronounced in men (9.7%/19.9%) than in women (29.5%/32.1%). MetS was slightly more prevalent in women compared to men, but this difference became smaller when using a BMI cut-off of 30 kg/m^2^ in both genders (table S1, see supporting information).

**Figure 2:**
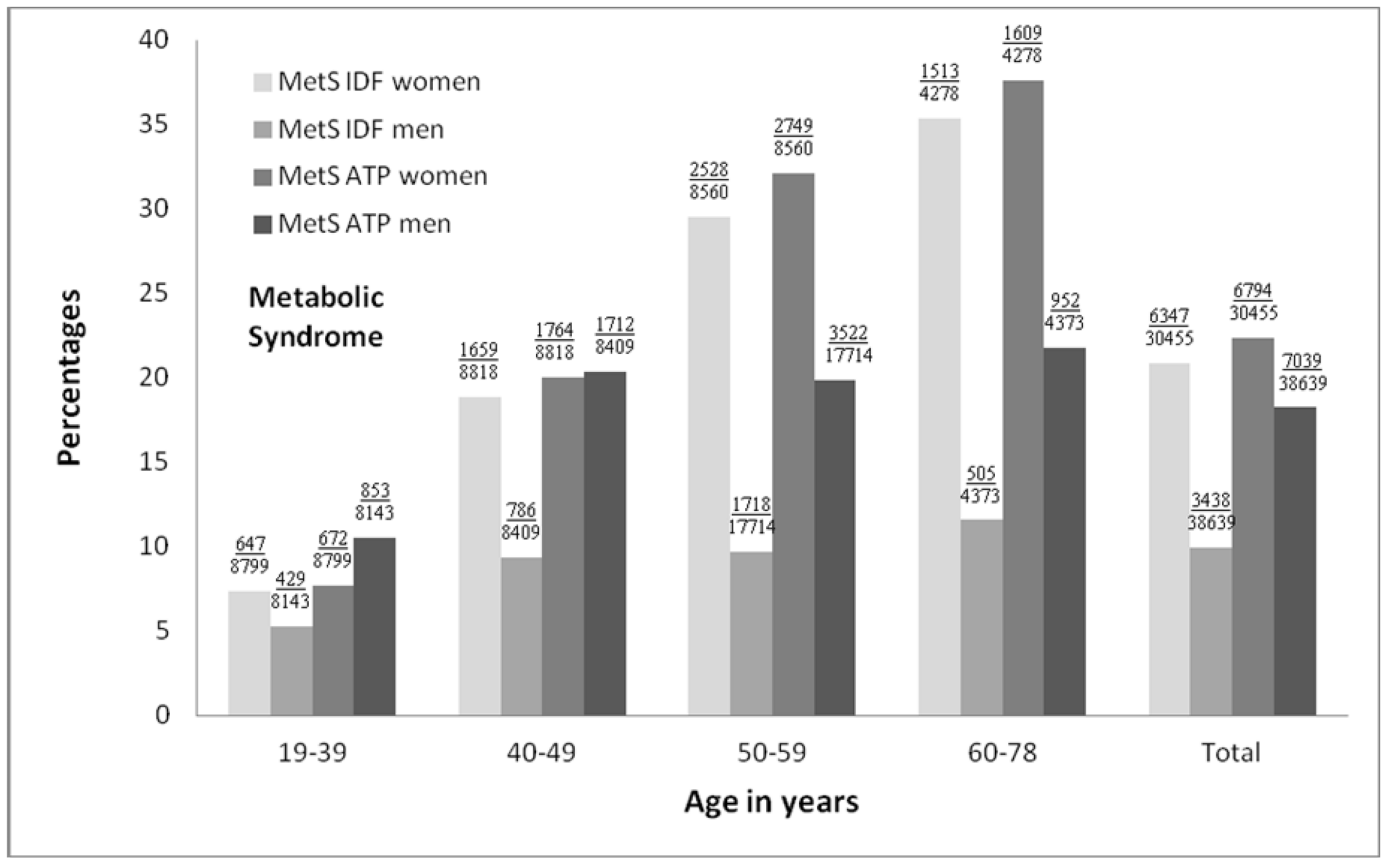
Frequency of MetS according to gender and age group. MetS indicates the metabolic syndrome; IDF, the International Diabetes Federation (MetS IDF) criteria and the National Cholesterol Education Program–Adult Treatment Panel III (MetS ATP) criteria. Numbers above each bar indicate total number of persons with MetS/total number of persons in the given age group; All *P*<0.0001 for each of the 4 MetS/gender combination across age groups. Within each age group, *P*<0.0001 between genders, except for MetS ATP in ages 40–49 years (*P* = 0.57).

Participants who had MetS defined by IDF also had MetS defined by NCEP-ATP III. However, of those participants who had MetS defined by NCEP-ATP III, 30% (4048/13833; 447 women and 3601 men) did not have MetS defined by IDF (data not shown) supporting the conclusion that the IDF definition is more restrictive than NCEP-ATP III, especially in men (figure 2).

Taking into account age group, the prevalence of MetS defined by both IDF and NCEP-ATP III significantly increased across ages for both genders (*P*<0.0001; figure 2). The increase in MetS prevalence from age group 19–39 years to 60–78 years was almost 5-fold (7.4%/7.6% to 35.4%/37.6%, for IDF/NCEP-ATP III, respectively) in women and 2-fold (5.3%/10.5% to 11.5%/21.8%) in men reflecting less increase in men older than 49 years. Within each age group, there was a significantly higher prevalence of MetS in women compared to men when using IDF, while for NCEP-ATP III this was only the case for women aged 50 years or above (all *P*<0.0001; figure 2).

### Outcome

**Figure 3:**
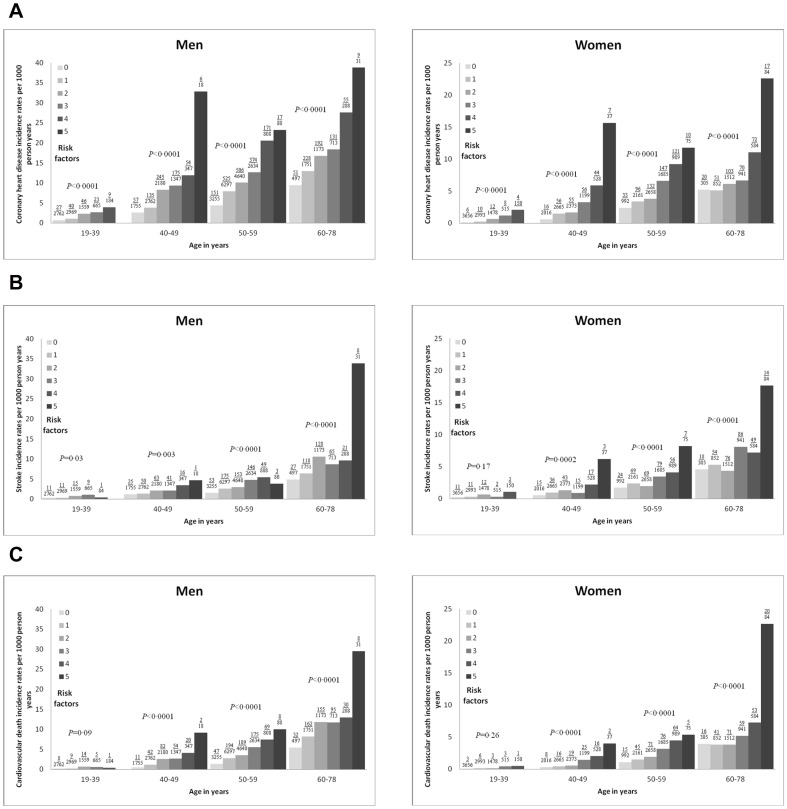
Age- and gender-specific incidence rates per 1000 person years for CVD with increasing number of risk factors. Risk factors refer to the components of the metabolic syndrome (MetS); and CVD, cardiovascular disease: (A) fatal and nonfatal CHD, (B) fatal and nonfatal stroke, and (C) cardiovascular death. Numbers above each bar indicate event/person. Overall trend with *P*<0.0001 for incidence rates with increasing number of MetS risk factors and age categories at baseline. Within each age group, the reported *P* values indicate significant differences in incidence rates with rising number of MetS risk factors, except in men (for CVD) and women (for stroke and CVD) below age 40 years.

During the average of 12.2 years of follow-up, the number of CHD, stroke, and CVD mortality in men/women were 3222/1146, 1189/768, and 1412/638, respectively (table S1, see supporting information). The incidence rates per 1000 person years for the 3 CVD events generally increased with rising number of components of MetS (*P*<0.0001) and age categories (*P*<0.0001) at baseline, with the strongest relations to CHD incidence rates and the weakest relationship with stroke and those below 40 years of age (figure 3). In women three or more elements, resembling MetS, had to be present to identify a significant risk increase (figure 3).

A comparison of men and women within each age group confirmed higher incidence rates in men for CHD in participants older than 39 years with 0–4 risk factors present, for CVD mortality in participants older than 49 years with 1–4 risk factors present, and for stroke in participants older than 49 years with 2–3 risk factors present (figure 3).

**Figure 4:**
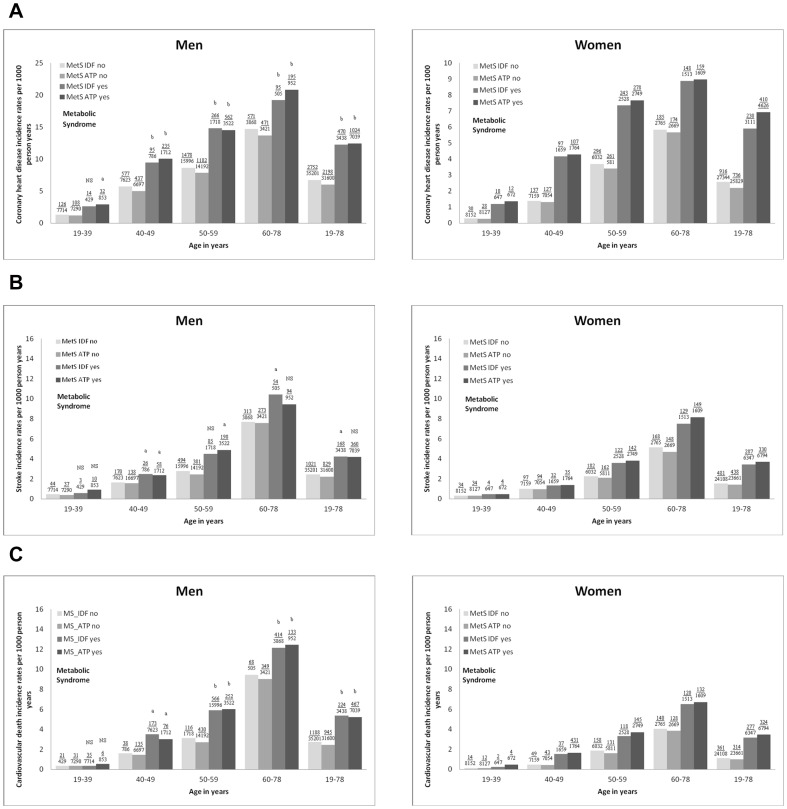
Age- and gender-specific incidence rates per 1000 person years for CVD with or without the presence of MetS. MetS IDF indicates the metabolic syndrome defined according to the International Diabetes Federation criteria; MetS ATP, the metabolic syndrome defined according to the National Cholesterol Eduation Program-Adult Treatment Panel III; and CVD, cardiovascular disease: (A) fatal and nonfatal CHD, (B) fatal and nonfatal stroke, and (C) cardiovascular death. Numbers above each bar indicate events/persons. Comparison of incidence rates between men and women with MetS within each age group using Pearson Chi^2^-test, *P*<0.05^a^, *P*<0.0001^b^, and NS indicates non-significance, *P*>0.05.

Even after adjusting for the known higher incidence rates with aging and male gender (*P*<0.0001; figure 4), MetS at baseline was associated with higher incidence rates especially for CHD and when MetS was defined by NCEP-ATP III. For stroke, the incidence rates were very low for those younger than 50 years with MetS, indicating that MetS was not strongly associated with stroke in younger participants.

The gender-specific HRs for CHD, stroke, and CVD mortality when baseline MetS was defined by either IDF or NCEP-ATP III (table 4) were significantly associated with all three CVD events (all *P*<0.0001), independent of age, total cholesterol, and smoking as well as fasting status, and comparable HRs were observed for both definitions of MetS. However, in women compared to men MetS defined by especially NCEP-ATPIII was closer associated with CHD risk (HRs 1.93/2.03 vs. 1.60/1.62 for IDF/NCEP-ATP III), with CVD mortality risk (HRs 1.77/2.06 vs. 1.73/1.65), and with stroke risk (HRs 1.58/1.77 vs. 1.51/1.53). Furthermore, whereas in men the HRs for a CVD event were independent of age (MetS*age, *P*>0.05; table 2), in women the HRs for CHD declined with age (from HRs 3.23/3.98 to 1.55/1.56; MetS*age, *P* = 0.01/*P* = 0.001 for IDF/NCEP-ATPIII) while the HRs for stroke tended to increase (from HRs 1.31/1.25 to 1.55/1.83; MetS*age, *P*>0.05).

**Table 4:** Hazard ratio for different definitions of the metabolic syndrome by age category and event type in men and women.

				Men							Women			
		MetS IDF			MetS ATP				MetS IDF			MetS ATP		
	N	HR	95%CI	*P*-value	HR	95%CI	*P*-value	N	HR	95%CI	*P*-value	HR	95%CI	*P*-value
**Coronary heart disease**														
19–39 years	140	1.46	0.84–2.55	0.18	1.50	0.99–2.27	0.05	40	3.23	1.51–6.89	0.0025	3.98	1.94–8.20	0.0002
40–49 years	672	1.59	1.28–1.98	<0.0001	1.65	1.40–1.95	<0.0001	234	2.56	1.96–3.35	<0.0001	2.66	2.04–3.48	<0.0001
50–59 years	1744	1.71	1.50–1.95	<0.0001	1.65	1.49–1.83	<0.0001	539	1.88	1.58–2.23	<0.0001	2.02	1.70–2.40	<0.0001
60–78 years	666	1.29	1.03–1.60	0.02	1.46	1.23–1.72	<0.0001	333	1.55	1.24–1.94	0.0001	1.56	1.25–1.94	<0.0001
19–78 years^a^	3222	1.60	1.45–1.77	<0.0001	1.62	1.50–1.75	<0.0001	1146	1.93	1.71–2.18	<0.0001	2.03	1.80–2.29	<0.0001
**Stroke**														
19–39 years	47	0.98	0.30–3.19	0.97	1.88	0.91–3.89	0.09	38	1.31	0.45–3.78	0.62	1.25	0.43–3.61	0.68
40–49 years	196	1.54	1.01–2.34	0.04	1.37	1.00–1.88	0.05	129	1.33	0.88–2.00	0.17	1.35	0.90–2.01	0.14
50–59 years	579	1.60	1.27–2.02	<0.0001	1.76	1.47–2.09	<0.0001	304	1.74	1.38–2.21	<0.0001	1.95	1.54–2.46	<0.0001
60–78 years	367	1.38	1.03–1.84	0.032	1.25	0.99–1.58	0.066	297	1.55	1.22–1.96	0.0003	1.83	1.44–2.32	<0.0001
19–78 years^a^	1189	1.51	1.28–1.78	<0.0001	1.53	1.35–1.73	<0.0001	768	1.58	1.36–1.84	<0.0001	1.77	1.52–2.05	<0.0001
**Cardiovascular death**														
19–39 years	37	0.72	0.17–3.03	0.66	1.05	0.42–2.63	0.91	16	1.21	0.26–5.61	0.81	2.90	0.88–9.56	0.08
40–49 years	211	2.17	1.52–3.10	<0.0001	1.70	1.27–2.27	0.0003	86	2.67	1.72–4.15	<0.0001	3.12	2.01–4.84	<0.0001
50–59 years	682	1.91	1.56–2.34	<0.0001	1.84	1.57–2.15	<0.0001	276	1.70	1.33–2.16	<0.0001	2.07	1.62–2.63	<0.0001
60–78 years	482	1.34	1.03–1.73	0.027	1.37	1.12–1.68	0.0022	260	1.60	1.25–2.06	0.0002	1.71	1.33–2.20	<0.0001
19–78 years^a^	1412	1.73	1.50–2.00	<0.0001	1.65	1.47–1.84	<0.0001	638	1.77	1.51–2.09	<0.0001	2.06	1.75–2.42	<0.0001

N indicates the number of events; MetS IDF, metabolic syndrome according to the International Diabetes Federation; and MetS ATP, metabolic syndrome according to the National Cholesterol Education Program – Adult Treatment Panel III.

Cox model adjusted for smoking (yes/no), cholesterol (continuous), and fasting (full/semi/no fasting).

a For the total population, the Cox model is adjusted for age, smoking (yes/no), total cholesterol (continuous), and fasting (full/semi/no fasting).

### Sensitivity Analyses

Replicating the above analyses in subsets using WC instead of BMI, and using a reduced dataset excluding participants in antihypertensive treatment and non-fasting or semi-fasting participants, generally showed similar trends. Worth noting is that the mean values of triglycerides, SBP, and DBP, as well as the prevalence/incidence rates of MetS and its components were attenuated. Furthermore, using WC instead of BMI showed a higher prevalence of MetS defined by IDF in men compared to women.

## Discussion

Using modified versions of the IDF- and the revised NCEP-ATP III criteria of MetS, the main findings of the present study were that the prevalence and prognostic significance of MetS at baseline showed great variations among countries and were influenced by both age and gender. With older age, the prevalence of MetS increased 5-fold in women and 2-fold in men (figure 2) and the pattern of the individual MetS components changed in women (obesity was surpassed by high SBP) but not in men in whom SBP was dominating in all ages (figure 1). Independently of age the prevalence of MetS based on the NCEP-ATP III criteria almost doubled that of IDF criteria in men whereas it was equal in women (figure 2). Incidence rates for all three CVD events increased with more components of MetS present independently of the type of CVD event, age and gender (figure 3), whereas the risk of a CVD event associated with MetS was (1) higher in women than in men especially when using the NCEP-ATP III criteria (table 4), and (2) independently of age in men whereas in women CHD risk decreased and stroke risk tended to increase with older age (table 4).

These results were reproducible in all sensitivity analyses, although attenuated. To the best of our knowledge this is the first study to examine the age and gender specific prevalence and prognostic significance of MetS using the IDF and the revised NCEP-ATP III criteria in a large European prospective cohorts study.

### Prevalence of MetS and its components

Our finding, that MetS prevalence increased with age, is consistent with previous work [3], [5], [8]–[9], [11], [28], [36]. Furthermore, the steeper age-related increase in MetS prevalence in women compared to men, has also been shown previously [5], [8], and may be attributable to the steep increase in BP in women after menopause which initiates a more rapid decrease in endothelial function. Consistently, we found a shift in the dominance of the individual MetS components with age, from obesity in younger women to elevated BP in older women. Similarly, Lawlor et al [12] found that the most prevalent component of MetS in postmenopausal women was elevated BP. The less steep increase in prevalence of MetS with older age in men, found in the present study, could partly be due to the dominance of the BP component in all age groups. The relative difference between the genders in components of MetS with older age has been shown previously [9].

Country variations in prevalence of MetS have also been found in other studies [5], [8], [9], [11], [17]–[20], [27]. For instance, comparing studies from Germany [5], Norway [8], and Greece [11] show variations in MetS prevalence from 9–16% in men below age 40 years to 34–45% in men between ages 60–69 years, and the corresponding prevalence in women was 5–8% and 35–46%. These age differences were slightly accentuated when using the IDF criteria. The country variation in the present study could not be explained by variations in age and gender indicating that it was due to some country specific differences in either genes, lifestyle and/or population selection.

### Incidence rates and prognostic importance of MetS and its components

Previously, it has been shown that incidence rates for CVD increase with more components of MetS present [36], [37], as well as with the presence of MetS [3], [5], [22]. Furthermore, some [17]–[22] but not all [2], [12] previous studies have shown a higher CVD event risk conferred by MetS in women compared to men. We confirmed and extended these findings by showing (1) that it was true for the risk of CHD, stroke, and CVD mortality, (2) that the risk associated with MetS was higher in women especially when using the NCEP-ATP III criteria, and (3) that the CVD event risk associated with MetS was independently of age in men whereas in women CHD risk decreased and stroke risk tended to increase with older age.

The CVD event risk associated with MetS can be explained by the fact that MetS includes both metabolic risk factors promoting atherosclerosis and CHD and hemodynamic risk factors promoting arteriosclerosis and stroke [38], [39]. In women the revised NCEP-ATP III criteria was more closely associated with outcome than MetS defined by the IDF criteria which might be explained by the fact that elevated BMI, which is mandatory for the IDF definition and very prevalent in women with MetS, is only weakly related to CVD outcome.

Furthermore, the weaker associations between CVD event risk and MetS defined by the IDF as well as NCEP-ATP III criteria found previously in women [2], [12] could be due to the lack of age-stratification as well as the lack of inclusion of younger women. The increased relative risk for CHD among young women with MetS might be explained by the low absolute risk of CHD in these young women. Indeed, we showed very low incidence rates per 1000 person years for CHD in women aged 19–39 years without MetS (figure 4A). Consistently, Gami et al [18] showed in a meta-analysis that the association between MetS and incident CVD events and death was stronger in studies enrolling lower risk individuals. The apparent age independency of MetS associated CVD event risk in men, can be explained by lower relative risk in younger men with MetS due to higher absolute risk in younger men without MetS, by higher relative risk in middle-aged men due to sufficient exposure time, and finally by a lower relative risk in older men due to selection bias. Alternatively, the reduction of HRs with aging for CHD and to some degree of CVD mortality in women with MetS might be due to increasing competing risk (i.e. cancer) [40], but that would not explain the gender difference. In contrast, the stroke risk associated with MetS seemed to increase with age in women. This age-related increase in HRs for stroke in women with MetS might reflect the increased contribution of SBP to the definition of MetS. As seen in figure 1, we showed that the frequency of the BP component of MetS increased from 20% in women aged 19–39 years to 80% in women aged 60–78 years as compared to 45% and 80% in men. Furthermore, from table 3B it is seen that the median SBP value increased significantly with age. Lastly, in previous work [41] we showed that the HR for SBP does not decrease with age, implying the importance of SBP also in the elderly.

### Strengths

The strength of the present investigation was the large sample size of 69 094 individuals, with a mean follow-up time of 12.2 years leading to a relatively large number of CVD events: 4368, 1957, 2050 incident CHD-, stroke- and CVD-events, respectively, which was more than in many previous studies, and which allowed robust sensitivity analyses. Other strengths were the inclusion of a wide age range, an almost equal proportion of men and women, and the standardized baseline and endpoint assessment available from the well-characterized MORGAM cohorts, with individual validation of the diagnosis in the majority of fatal and nonfatal events.

### Limitations

Some limitations should also be considered. The baseline age distribution of the various populations differed, and therefore any age difference observed may also reflect differences between the populations. We tried to minimize the influence of the populations by adjusting for country in the Cox model. Furthermore, when comparing MetS prevalence among the populations, we used a fixed age group, which was covered by all populations. However, since most populations are from age 25 years and onwards, our results for the youngest age group should be interpreted with caution due to lack of statistical power to detect associations.

The MetS status of the participants, which is bound to change during the follow-up period, was available only at baseline, causing likely attenuation of the estimates of the hazard ratios.

Since data on plasma glucose was not available, the self-reported presence of diabetes or use of anti-diabetic drugs was used instead. Therefore, we have underestimated the prevalence of diabetes. Although most of the “missed” cases would be defined as having MetS on account of the coexistence of other components of MetS, some might have been “missed” probably leading to an underestimation of the HR associated with MetS because subjects with diabetes generally are at high CVD risk. Therefore, we do not believe that the underestimation of MetS influenced the prognostic interactions between age, gender and the risk of MetS. Although we used BMI instead of WC in order to maximize sample size, this was in line with other studies [6], [9], [17]–[20]. In a meta-analysis by Gami et al [18] it was shown that substitution of BMI for WC or waist-to-hip ratio in NCEP-based criteria did not appear to affect the results. Moreover, since fasting levels differed between cohorts, a categorized fasting variable was used as adjustment in the Cox model. In both of these cases, we carried out sensitivity analyses using WC instead of BMI, or including only full-fasting individuals, and showed similar, although slightly attenuated results. For the latter case, Hildrum et al [8] showed no statistically significant difference between the fasting and the non-fasting samples in the prevalence of the IDF- or ATP-proxies of MetS. Finally, our findings may not apply to non-Europeans or patients with CVD because the latter were excluded.

## Conclusion

MetS was prevalent and associated with increased incidence of CHD, stroke, and CVD mortality. However, prevalence and prognostic importance were strongly dependent on age and gender, which seemed to account for some of the differences between countries. The CHD risk associated with MetS was higher in women especially when using the NCEP-ATP III criteria and it was independently of age in men whereas in women CHD risk decreased significantly and stroke risk tended to increase with older age. These results emphasise the importance of being critical of MetS in its current form as a marker of CVD especially in women, and advocate for a redefinition of MetS taking into account age especially in women.

## Supporting Information

Table S1 **Characteristics of the MORGAM cohorts included in the analyses.**
(DOC)

Text S1 **Appendix.**
(DOC)
