# Correction: Impact of Age and Gender on the Prevalence and Prognostic Importance of the Metabolic Syndrome and Its Components in Europeans. The MORGAM Prospective Cohort Project

**DOI:** 10.1371/journal.pone.0128848

**Published:** 2015-05-15

**Authors:** Julie K. K. Vishram, Anders Borglykke, Anne H. Andreasen, Jørgen Jeppesen, Hans Ibsen, Torben Jørgensen, Luigi Palmieri, Simona Giampaoli, Chiara Donfrancesco, Frank Kee, Giuseppe Mancia, Giancarlo Cesana, Kari Kuulasmaa, Veikko Salomaa, Susana Sans, Jean Ferrieres, Jean Dallongeville, Stefan Söderberg, Dominique Arveiler, Aline Wagner, Hugh Tunstall-Pedoe, Wojciech Drygas, Michael H. Olsen, MORGAM Project

There is an error in the affiliation for the 11th author. The correct affiliation for Giuseppe Mancia is: Istituto Auxologico Italiano and University of Milano-Bicocca, Milan, Italy.
