# Supplementary material for: Impact of Age and Gender on the Prevalence and Prognostic Importance of the Metabolic Syndrome and Its Components in Europeans. The MORGAM Prospective Cohort Project
Source: PLoS One. 2014 Sep 22;9(9):e107294. doi: 10.1371/journal.pone.0107294 (PMC4171109; doi:10.1371/journal.pone.0107294)
Supplement: Table S1 — Characteristics of the MORGAM cohorts included in the analyses. (DOC) [file pone.0107294.s001.doc]

| **Table S1 Characteristics of the MORGAM cohorts included in the analyses** | | | | | | | | | | | |  |
| --- | --- | --- | --- | --- | --- | --- | --- | --- | --- | --- | --- | --- |
| **Country** | **Population** | **Type of Cohorts** | **Age Range at Baseline^a^** | **Survey Period** | **Years of Follow-Up** | **Total Years of Observa-tion for CHD^b^** | **No. of Subjects^c^** | **% of MetS IDF^d^** | **% of MetS NCEP-ATP III^d^** | **No. of CHDs** | **No. of Strokes** | **No. of CVDs** |
|  |  | **(No. of Cohorts)** |  |  |  |  | **M / W** | **M / W / W^1^** | **M / W / W^1^** | **M / W** | **M / W** | **M / W** |
| Denmark | DEN-GLO | MONICA centre (3) | 30, 40,  50, 60 | 1982-1992 | 9-19 | 95 500 | 3207 / 3264 | 7∙9 / 11∙3 / 5∙2 | 14∙0 / 12∙6 / 8∙4 | 334 / 132 | 162 / 125 | 170 / 76 |
|  | Glostrup |  |  |  |  |  |  |  |  |  |  |  |
| Finland | FIN-EAS | MONICA centre and MONICA procedures (4) | 25-64 | 1982-1997 | 11-26 | 86 438 | 3315 / 3743 | 18∙1 / 32∙9 / 20∙1 | 27∙5 / 34∙4 / 27∙9 | 278 / 119 | 121 / 79 | 84 / 34 |
|  | Eastern Finland and Oulu |  | 25-74 |  |  |  |  |  |  |  |  |  |
|  | FIN-WES | MONICA centre and MONICA procedures (4) | 25-64 | 1982-1997 | 11-26 | 75 067 | 2845 / 3076 | 16∙9 / 27∙1 / 14∙4 | 27∙2 / 27∙7 / 20∙0 | 198 / 82 | 98 / 70 | 76 / 42 |
|  | Turku-Loimaa and Helsinki |  | 25-74 |  |  |  |  |  |  |  |  |  |
| Sweden | SWE-NSW | MONICA centre (3) | 24-65 | 1986-1994 | 5-14 | 24 953 | 1441 / 1539 | 9∙9 / 27∙8 / 8∙6 | 21∙5 / 29∙8 / 16∙2 | 44 / 13 | 26 / 19 | 16 / 10 |
|  | Northern Sweden |  | 24-74 |  |  |  |  |  |  |  |  |  |
| Ireland | UNK-BEL | MONICA procedures, men only (1) | 49-60 | 1991-1994 | 10 | 23 389 | 2537 | 8∙7 | 21∙4 | 226 | 53 | 58 |
|  | Belfast |  |  |  |  |  |  |  |  |  |  |  |
| Scotland | UNK-EDI | MONICA procedures (1) | 25-64 | 1986 | 19 | 21 898 | 628 / 596 | 6∙3 / 21∙2 / 4∙5 | 13∙1 / 22∙9 / 10∙1 | 88 / 34 | 35 / 22 | 43 / 16 |
|  | Edinburgh |  |  |  |  |  |  |  |  |  |  |  |
|  | UNK-GLA | MONICA centre (4) | 25-64,  25-75 | 1986-1995 | 10-19 | 61 050 | 2266 / 2468 | 13∙5 / 36∙1 / 15∙4 | 26∙6 / 41∙3 / 28∙0 | 287 / 191 | 127 / 113 | 168 / 100 |
|  | Glasgow |  |  |  |  |  |  |  |  |  |  |  |
|  | UNK-SHH | MONICA procedures (1) | 39-59 | 1984-1987 | 18-21 | 144 832 | 4143 / 4163 | 9∙9 / 33∙0 / 12∙1 | 34∙5 / 38∙9 / 27∙2 | 793 / 407 | 248 / 169 | 358 / 186 |
|  | Nationwide |  |  |  |  |  |  |  |  |  |  |  |
| France | FRA-LIL | PRIME (MONICA procedures), men only (1) | 49-60 | 1991-1993 | 10 | 22 018 | 2336 | 8∙0 | 15∙6 | 146 | 38 | 31 |
|  | Lille |  |  |  |  |  |  |  |  |  |  |  |
|  | FRA-STR | PRIME (MONICA procedures), men only (1) | 49-60 | 1991-1993 | 10 | 21 911 | 2337 | 11∙3 | 19∙0 | 144 | 24 | 23 |
|  | Strasbourg |  |  |  |  |  |  |  |  |  |  |  |
|  | FRA-TOU | PRIME (MONICA procedures), men only (1) | 49-60 | 1991-1993 | 10 | 22 617 | 2420 | 5∙0 | 10∙8 | 154 | 22 | 14 |
|  | Toulouse |  |  |  |  |  |  |  |  |  |  |  |
| Italy | ITA-BRI | MONICA centre (3) | 25-66 | 1986-1994 | 9-16 | 54 289 | 2206 / 2297 | 8∙1 / 18∙7 / 9∙3 | 13∙9 / 20∙3 / 12∙7 | 142 / 40 | 39 / 21 | 45 / 23 |
|  | Brianza |  |  |  |  |  |  |  |  |  |  |  |
|  | ITA-PAM | MONICA procedures (1) | 25-75 | 1990-1993 | 9-12 | 20 089 | 936 / 952 | 6∙5 / 14∙8 / 7∙4 | 16∙9 / 15∙7 / 11∙1 | 51 / 10 | 24 / 14 | 22 / 5 |
|  | Pamela |  |  |  |  |  |  |  |  |  |  |  |
|  | ITA-ROM | MONICA centre and MONICA procedures (3) | 19-78 | 1983-1995 | 8-19 | 114 104 | 3736 / 4220 | 15∙3 / 45∙0 / 29∙1 | 22∙4 / 46∙1 / 35∙5 | 199 / 64 | 108 / 100 | 169 / 107 |
|  | Area Latina |  |  |  |  |  |  |  |  |  |  |  |
| Spain | SPA-CAT | MONICA centre and MONICA procedures (1) | 25-68 | 1986-1988 | 10-12 | 23 397 | 1220 / 1258 | 5∙1 / 26∙4 / 13∙9 | 11∙1 / 27∙3 / 19∙0 | 33 / 11 | 32 / 24 | 16 / 4 |
|  | Catalonia |  |  |  |  |  |  |  |  |  |  |  |
| Poland | POL-WAR | MONICA centre (3) | 34-65 | 1983-1993 | 6-16 | 27 132 | 2331 / 2326 | 11∙4 / 29∙2 / 16∙4 | 16∙3 / 30∙4 / 21∙9 | 94 / 36 | 25 / 11 | 107 / 31 |
|  | Warsaw |  |  |  |  |  |  |  |  |  |  |  |
| Russia | RUS-NOV | MONICA centre and MONICA procedures (1) | 24-65 | 1994-1995 | 4-5 | 4985 | 735 / 553 | 7∙2 / 39∙8 / 27∙3 | 13∙1 / 40∙6 / 32∙8 | 11 / 5 | 7 / 1 | 12 / 4 |
|  | Novosibirsk |  |  |  |  |  |  |  |  |  |  |  |
| All MORGAM Cohorts | |  |  |  |  | 843 668 | 38639 / 30455 | 9∙7 / 29∙5 / 14∙6 | 19∙9 / 32∙1 / 22∙8 | 3222 / 1146 | 1189 / 768 | 1412 / 638 |
| CHD indicates fatal and nonfatal coronary heart disease; stroke, fatal and nonfatal strokes; CVD, cardiovascular disease mortality; MetS IDF, metabolic syndrome according to the International Diabetes Federation criteria; MetS NCEP-ATPIII, metabolic syndrome according to the National Cholesterol Eduation Program - Adult Treatment Panel III criteria; M, men using a BMI (kg/m^2^) cut-off of 30 in the MS definition; W, women using a BMI cut-off of 25; and W^1^, women using a BMI cutt-off of 30. | | | | | | | | | | | | |
| ^a^The age range was continuous except in DEN-GLO, where the age was around 30, 40, 50 or 60 years at baseline. Three cohorts in FIN-EAS, FIN-WES, and SWE-NSW used the age range 25-64 years, while one cohort used the age range 25-74 years. | | | | | | | | | | | | |
| ^b^Analysis data set. | | | | | | | | | | | | |
| ^c^Number of subjects used in the analyses. | | | | | | | | | | | | |
| ^d^Due to differences in baseline age distribution among the different populations, the prevalence of the metabolic syndrome is presented for a fixed age-interval of 50-59 years, allowing for a meaningful comparison between the populations. | | | | | | | | | | | | |
