# Supplementary material for: Impact of Age and Gender on the Prevalence and Prognostic Importance of the Metabolic Syndrome and Its Components in Europeans. The MORGAM Prospective Cohort Project
Source: PLoS One. 2014 Sep 22;9(9):e107294. doi: 10.1371/journal.pone.0107294 (PMC4171109; doi:10.1371/journal.pone.0107294)
Supplement: Text S1 — Appendix. (DOC) [file pone.0107294.s002.doc]

**Text S1 Appendix**

Sites and key personnel of contributing MORGAM Centres.

**Denmark**

Glostrup, Capital Region, Research Centre for prevention and Health, Glostrup University Hospital: T. Jørgensen (principal investigator), C. Agger, A. Borglykke, M. Olsen;

**Finland**

FINRISK, National Institute for Health and Welfare (THL), Helsinki: V. Salomaa (principal investigator), A. Juolevi, E. Vartiainen, P. Jousilahti;

MORGAM Data Centre, National Institute for Health and Welfare (THL), Helsinki: K. Kuulasmaa (head), Z. Cepaitis, A. Haukijärvi, B.Joseph, J.Karvanen, S. Kulathinal, M. Niemelä, O. Saarela;

**France**

National Coordinating Centre, National Institute of Health and Medical Research

(U258), Paris: P. Ducimetière (national coordinator), A. Bingham;

PRIME/Strasbourg, Department of Epidemiology and Public Health, University of Strasbourg, Faculty of Medicine, Strasbourg: D. Arveiler (principal investigator), B. Haas, A. Wagner;

PRIME/Toulouse, Department of Epidemiology, Faculty of Medicine, Toulouse-

Purpan, Toulouse: J. Ferrières (Principal Investigator), J-B. Ruidavets and V. Bongard;

PRIME/Lille, Department of Epidemiology and Public Health, Pasteur Institute of

Lille: P. Amouyel (principal investigator), M. Montaye, J. Dallongeville;

**Italy**

National Coordinating Centre MORGAM, Dipartimento de Medicina Sperimentale, Università degli Studi dell’Insubria, Varese: M. Ferrario (national coordinator), P. Chiodini, S. Sarman;

Brianza, Dipartimento de Medicina Sperimentale, Università degli Studi dell’Insubria, Varese: M. Ferrario (principal investigator); Dipartimento de Medicina, Prevenzione e Biotecnologie Sanitarie, Università degli Studi Milano-Bicocca, Monza: G. Cesana (principal investigator), C. Fornari;

Pamela, Dipartimento de Medicina, Prevenzione e Biotecnologie Sanitarie, Università degli Studi Milano-Bicocca, Monza: R. Sega, G. Mancia, R. Facchetti;

Area Latina: Unit of Epidemiology of Cerebro and Cardiovascular Diseases, National Centre for Epidemiology, Surveillance and Health Promotion, Istituto Superiore di Sanità, Rome: S. Giampaoli, L. Palmieri (principal investigators), C. Donfrancesco;

**Poland**

Warsaw, Department of Cardiovascular Epidemiology and Prevention, National Institute of Cardiology, Warsaw: G. Broda (principal investigator), P. Kurjata, S.L. Rywik, M. Polakowska, A. Pytlak;

Krakow, Unit of Clinical Epidemiology and Population Studies, School of Public Health,

Jagiellonian University, Krakow: A. Pająk (principal investigator), E. Kawalec;

**Russian Federation**

Novosibirsk, Institute of Internal Medicine, Siberian Branch of the Russian Academy of Medical Science, Novosibirsk: Y. Nikitin (principal investigator), S. Malyutina, V. Gafarov, V. Feigin, G. Siminova, M. Voevoda, T. Vinogradova, N. Nasonova, E. Veriovkin;

**Spain**

Institute of Health Studies, Barcelona: Susana Sans (principal investigator); I. Balaguer-Vintró (former principal investigator), L. Balañá, G. Paluzie;

**Sweden**

Northern Sweden, Umeå University Hospital, Medicine, Umeå: M. Eriksson, S. Söderberg (principal investigator) B. Stegmayr (former principal investigator), K. Asplund (former principal investigator);

**United Kingdom**

PRIME/Belfast, Queen´s University Belfast, Belfast, Norhern Ireland: F. Kee (principal investigator);

MORGAM Coordinating Centre, Queen's University Belfast, Belfast, Northern

Ireland: A. Evans, S. Cashman;

**MORGAM Management Group**:

K. Kuulasmaa (Helsinki, Finland), S. Blankenberg (Hamburg, Germany), M. Perola (Helsinki, Finland), A. Evans (Chair, Belfast, United Kingdom), M. Ferrario (Varese, Italy), F. Kee (Belfast, United Kingdom), A. Palotie (Hinxton, United Kingdom), A. Peters (Neuherberg, Germany), V. Salomaa (Helsinki, Finland), D. Tregouet (Paris, France), H. Tunstall-Pedoe (Dundee, United Kingdom). Previous members: K. Asplund (Stockholm, Sweden), L. Peltonen (Helsinki, Finland), D. Shields (Dublin, Ireland), B. Stegmayr (Umeå, Sweden), P-G. Wiklund (Umeå, Sweden).
